# Diagnostic performance and reliability of robotic ultrasonography and artificial intelligence-driven synovitis assessment in rheumatoid arthritis: results from the Controlled ARTHUR Trial

**DOI:** 10.1136/rmdopen-2025-006099

**Published:** 2025-11-11

**Authors:** Mads Ammitzbøll-Danielsen, Mikkel Østergaard, Lydia Tamm, Lene Terslev

**Affiliations:** 1 Copenhagen Center for Arthritis Research, Rigshospitalet Center for Rheumatology and Spine Diseases, Copenhagen, Denmark; 2 Department of Clinical Medicine, University of Copenhagen Faculty of Health and Medical Sciences, Copenhagen, Denmark; 3 Department of Medicine and Therapeutics, The Chinese University of Hong Kong Faculty of Medicine, Hong Kong, Hong Kong

**Keywords:** Ultrasonography, Arthritis, Rheumatoid, Synovitis, Outcome Assessment, Health Care, Machine Learning

## Abstract

**Aim:**

To evaluate the reliability and diagnostic performance of artificial intelligence (AI) driven robotic ultrasonography (RUS) compared with human ultrasonography (HUS) performed by a trained rheumatologist for assessing synovitis in the hands of healthy controls and patients with rheumatoid arthritis (RA).

**Methods:**

20 healthy controls and 29 patients with RA eligible for initiation or intensification of disease-modifying anti-rheumatic drugs, with at least one clinically swollen joint in the hands, were included. The ultrasound robot scanned controls and patients twice without interval, whereas HUS was performed once. Synovitis was scored from 0 to 3 for Greyscale (GS) and Doppler (CD) by RUS (using AI software) and HUS according to the European League Against Rheumatism-OMERACT scoring system (range 0–66).

Intrarobot and human-robot reliability and agreement were assessed. Furthermore, the diagnostic performance of RUS for detecting arthritis was evaluated using the clinical arthritis diagnosis as reference.

**Results:**

Intrarobot reliability was moderate to good, with intraclass correlation coefficients (ICCs) values of 0.65 (GS) and 0.86 (CD) in patients with RA. Human-robot agreement was moderate, with ICCs of 0.59 (GS) and 0.64 (CD). At joint level, RUS obtained higher scores for MCP joints than HUS, while scores were comparable for other joint groups. The diagnostic accuracy of RUS for detecting clinically-detected arthritis was 59%.

**Conclusion:**

A moderate to good overall agreement was seen between RUS and HUS in assessing synovitis in RA hands. However, at the joint level, low agreement was observed, particularly for MCP joints in both intrarobot and human-robot comparisons, which affected diagnostic performance.

**WHAT IS ALREADY KNOWN ON THIS TOPIC**

**WHAT THIS STUDY ADDS**

- This investigator-initiated study provides important information on the agreement between robotic ultrasonography and human-performed ultrasound for the assessment of synovitis and diagnostic accuracy with the clinical diagnosis as reference standard, in healthy controls and RA patients with joint inflammation in the hands.

**HOW THIS STUDY MIGHT AFFECT RESEARCH, PRACTICE OR POLICY**

- This study clearly demonstrates the great potential of robotic ultrasonography, while also highlighting that the current versions of ARTHUR/Diana (1.5.1/2.0.1) are not yet ready for implementation in daily clinical practice.

## Introduction

 Rheumatoid arthritis (RA) is the most common inflammatory arthritis.^1 2^ A key feature of RA is synovitis, particularly in the wrist, metacarpophalangeal (MCP) and proximal interphalangeal (PIP) joints.^3^ If left untreated, synovitis can lead to significant joint damage and disability. Early diagnosis is therefore crucial for achieving optimal treatment outcomes, emphasising the need for sensitive assessment methods.^4^

Ultrasound has been established as a reliable tool for assessing synovitis and is recommended in the diagnostic process to facilitate early detection, as described in the European League Against Rheumatism (EULAR) guidelines for the use of imaging of the joints in the clinical management of RA.^5^ Studies have shown that systematic ultrasound examination of the hands and feet can shorten the diagnostic process, leading to earlier intervention.^6 7^ However, limited access to ultrasound specialists and resource constraints may delay the access and hence the diagnosis.

To address this challenge, the Arthritis Ultrasound Robot (ARTHUR) has been developed as an innovative, CE-marked automated system designed to perform ultrasound scans of hand and wrist joints.^8^ When integrated with an artificial intelligence (AI)-driven Diagnosis Aid Network for Rheumatoid Arthritis (DIANA) software system for scoring the images, ARTHUR provides a feasible method for assessing and quantifying synovitis.^8^

This study aimed to evaluate the reliability of ARTHUR (V.1.5.1) in combination with DIANA (V.2.0.1) in the assessment of synovitis. The primary objective was to assess the intrarobot agreement for robotic ultrasonography (RUS). The secondary objective was to assess agreement between RUS and standard human ultrasonography (HUS) as performed by a trained rheumatologist in detecting synovitis in the wrist and finger joints of healthy individuals and patients with RA and finally, to evaluate the diagnostic performance of RUS compared with HUS in these cohorts.

## Patients and methods 

### Patients and study design

Patients with RA, aged ≥18 years and eligible for initiation or intensification of conventional synthetic or biological disease-modifying anti-rheumatic drugs (DMARDs), with at least one clinically swollen joint in the hand, were recruited from the rheumatology outpatient clinic at Rigshospitalet, VHV, Copenhagen, Denmark, as part of the ARTHUR trial. In addition, healthy controls were included, recruited from hospital staff, with no clinically swollen or painful joints in the hands. Patients and healthy controls who had had surgery in the hands within 4 weeks before study initiation or who had severe deformity in the hands or wrists were excluded from participation. The sample size was determined based on findings from prior research in a related field.

### Robotic ultrasonography

**Figure 1:**
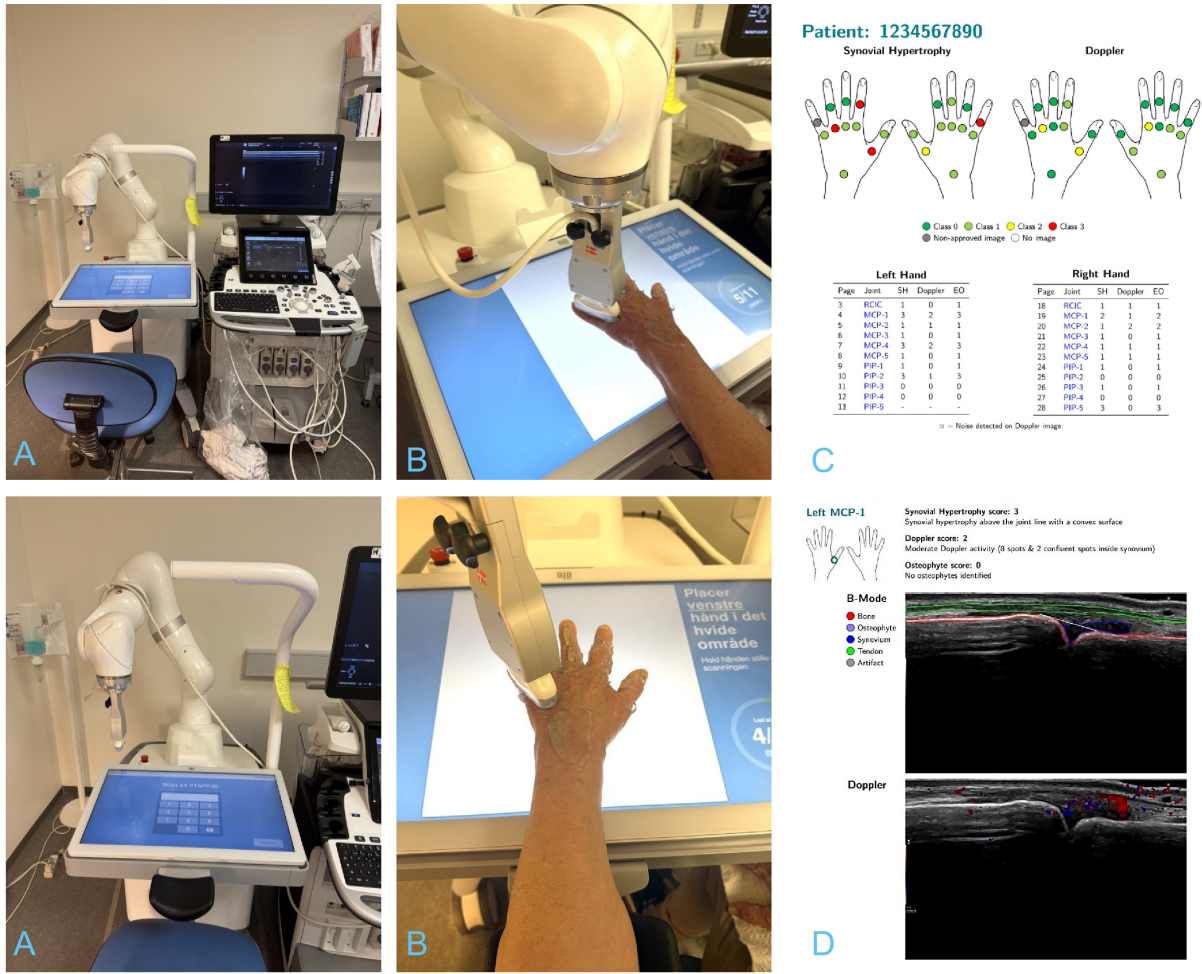
Robotic ultrasound assessment of hand joints. The Arthritis Ultrasound Robot connects with the ultrasound unit (**A**). The robot performs an ultrasound assessment of the patient (**B**). The report generated by the Diagnosis Aid Network for Rheumatoid Arthritis software system is presented to the clinician after the scanning (**C**). Manual visual inspection of all joints is possible, as illustrated for the left metacarpophalangeal joint 1 (**D**). MCP, metacarpophalangeal; PIP, proximal interphalangeal; EO, Global OMERACT-EULAR Synovitis Score; RCIC, Radiocarpal–Intercarpal joint; SH, Synovial hypertrophy.

ARTHUR is an automated system featuring a robotic arm and an integrated camera that precisely identifies joint regions in the hand.^8^ It independently positions a standard ultrasound probe, connected to an ultrasound unit, over the targeted joint and captures high-quality ultrasound images (figure 1). In this study, the GE Logiq E10 system equipped with a high-frequency linear probe (6–15 MHz) was used to ensure high sensitivity. Greyscale (GS) and colour Doppler (CD) settings were optimised by the provider of Arthur to ensure optimal performance and were consistently applied throughout the study for both RUS and HUS. The chosen GS map was B, Doppler frequency was 10 MHz, the pulse repetition frequency was 0.8 and the wall filter was 86.

ARTHUR is designed for self-guided operation, providing digital voice instructions to assist users through the scanning process. However, to ensure optimal scanning conditions for reliable comparison with HUS, a trained study nurse assisted with all scans, ensuring correct hand positioning and appropriate gel application. Furthermore, the same conditions were maintained for all scans, including identical room lighting, robot position and ultrasound machine parameters. Additionally, all conditions were carefully monitored by the provider of ARTHUR prior to study initiation and through periodic inspections during the study.

In the study, ARTHUR performed ultrasound assessments of the wrist (intercarpal position), the first–fifth MCP joints, the first interphalangeal joint (IP) and the second–fourth PIPs in both hands.

Synovitis was scored in the standard position using the AI-driven model DIANA based on the validated EULAR-OMERACT Synovitis Scoring system.^9 10^ GS and CD were assessed on a semiquantitative scale from 0 to 3. To evaluate intra-robot reliability, ARTHUR performed two consecutive scans on each patient and healthy control without an interval in between.

### Human ultrasonography

For HUS, a GE Logiq E10 ultrasound unit with a high-frequency linear ML 6–15 probe was used, maintaining the same GS and CD settings as those applied for the ARTHUR scanning throughout the study. Synovitis was assessed and scored in standard position using both GS and CD, following the semiquantitative OMERACT-EULAR Synovitis Scoring system^9 10^ assessment, which was performed by a single expert in musculoskeletal ultrasound who had demonstrated high intrareader and inter-reader agreement,^11 12^ but was not blinded to the clinical diagnosis. Each joint was evaluated individually, and total GS and CD scores at patient level were subsequently calculated (range 0–66 per modality).

### Definitions of synovitis and arthritis

We applied the following definitions for clinical and US-detected synovitis and arthritis.

Joint level

- Clinically-detected synovitis: Joints that are clinically swollen.
- US-detected synovitis: joints with GS>1 or GS≥1 combined with CD>1.

Patient level

- Clinically detected arthritis: diagnosis of RA and ≥1 clinically swollen joint in the hands.
- US-detected arthritis: at least one joint with GS >1, or GS ≥1 combined with CD>1 in the hands.

These definitions were used to assess the diagnostic value of the RUS and hence the clinical relevance.

### Clinical assessments

Disease activity was assessed at baseline using the Disease Activity Score-28 (DAS28),^13^ including the 28 swollen joint count (SJC), 28 tender joint count (TJC), and 22 SJC-hand (corresponding to the hand joints assessed by ultrasound).

### Statistical analysis

Descriptive statistics were used to summarise baseline characteristics. Agreement between RUS and HUS assessments was evaluated using the following methods:

Intrarobot reliability: agreement between the first (RUS1) and second (RUS2) robotic scans.

Human-robot agreement: agreement between RUS (both RUS1 and RUS2) and HUS.

At joint level, sum scores for each joint type were used and at patient level, a sum score for all joints was used, to assess percentage of exact agreement (PEA) and percentage of close agreement (PCA), defined as ±1. PEA was used to directly measure the frequency of identical ratings, providing a simple indicator of rater agreement, while PCA was applied to assess the proportion of measurements considered sufficiently similar for the purposes of our analysis.

Reliability was assessed using single measure intraclass correlation coefficients (ICCs). ICC values of <0.5, ≥0.5–0.74, ≥0.75–0.89 and ≥0.9 are indicative of poor, moderate, good and excellent reliability, respectively.^14^ As a sensitivity analysis, mean weighted (w) Kappa coefficients for all pairs were added to assess intrarobot agreement.^15^ The interpretation of Cohen’s kappa is: ≤ 0 indicates no agreement, 0.01–0.20 slight, 0.21–0.40 fair, 0.41–0.60 moderate, 0.61–0.80 substantial and 0.81–1.00 almost perfect agreement^16^

Sensitivity, specificity and accuracy were calculated for clinically-detected arthritis as the reference standard. Agreement for dichotomous outcomes was assessed using Cohen’s kappa.^16^

In the case of missing data at joint level, the joints were excluded from the analysis.

## Results

### Patient and healthy control characteristics

20 healthy controls (14 females, 6 males) and 29 patients with RA (21 females, 8 males) were included consecutively from April to December 2024 from the rheumatology outpatient clinic. Patients with RA were either initiating biological DMARDs or starting/escalating conventional synthetic DMARDs. Demographics and inclusion characteristics of the healthy controls and patients are presented in table 1. The median age (25th–75th percentile) was 60 years (57; 69) for those with RA and 40.5 years (34; 52) for healthy controls.

**Table 1:** Demographics, clinical and laboratory characteristics of RA patients and healthy controls

	RA patients (n=46)	Healthy controls (n=11)
Age (years)	60 (57;69)	40.5 (34;52)
Male, n (%)	8 (26.7)	6 (20.0)
Disease duration <1 year, n (%)	13 (43.3)	–
IgM-RM positive, n (%)	21 (70.0)	–
Anti-CCP positive, n (%)	22 (73.3)	–
CRP (mg/L)	4 (2.4;7.0)	–
Swollen joint count (0–28)	4 (2.50;5.5)	0 (0;0)
Tender joint count (0–28)	6 (3,5;7.5)	0 (0;0)
Swollen joint count hands (0–22)	3 (2;6)	
DAS28CRP	4.2 (3.8;4.8)	–
HAQ (0–3)	0.7 (0.75;1.375)	–
VAS Global (mm) (0–100)	64 (46;76)	–
Initiation or escalation of csDMARDs, n (%)*	15 (50.0)	–
Initiation of bDMARDs, n (%)*	15 (50.0)	–

Data are shown as median (IQR) or number (percentage) as appropriate.

* Methotrexate: n=15, TNF-alpha inhibitors: n=12, tocilizumab: n=1, filgotinib: n=1

Anti-CCP, anti-cyclic citrullinated peptide; bDMARD, biological disease-modifying anti-rheumatic drug; CRP, C reactive protein; csDMARD, synthetic disease-modifying anti-rheumatic drug; DAS28, Disease Activity Score for 28 joints; HAQ, health assessment questionnaire; IgM-RM, immunoglobulin M rheumatoid factor; RA, rheumatoid arthritis; VAS global, patient global visual analogue scale.

### Sum scores in patients and healthy controls (patient level)

In patients with RA, the GS median sum score in RUS round 1 (range 0–66) was 18 (14; 21) and for CD, 7 (5; 13), while in RUS 2 the GS median sum score was 19 (15; 21) and for CD: 7 (5; 13). The corresponding HUS values were GS median sum score 10 (7; 12.5) and for CD: 5 (3; 8). The median DAS28 was 4.3 (3.8; 4.8), SJC28: 4 (2.5; 5.5), TJC28: 6 (3.5; 7.5) and SJC22: 3 (2;6), see table 2.

**Table 2:** Patient level and joint region level score for healthy controls and RA patient for R (RUS 1 and RUS 2) and HUS

		Greyscale ultrasound			Colour Doppler ultrasound		
		RUS 1	RUS 2	HUS	RUS 1	RUS 2	HUS
Healthy controls							
Total PIP score	Median (25 p;75 p)	4 (3;5.25)	2.5 (2;4)	0 (0;0.25)	1.5 (0;3)	1 (0.75;2)	0 (0;0)
	Mean±SD	5.2±4.2	0.3.75±4.1	0.45±1.0	1.75±2.0	1.7±1.9	0.1±0.3
Total MCP score	Median (25 p;75 p)	11(10;13.25)	11(9;12.3)	2.5 (1;4)	4 (2.75;6)	3 (1.75;4)	0.5 (0;0)
	Mean±SD	11.6±3.1	10.8±2.1	3.1±2.5	4.1±2.2	3.1±2.2	0.05±0.2
Total Wrist Score	Median (25 p;75 p)	2 (0.75;2)	2 (1;3)	0 (0;1)	0 (0;1)	0 (0;1)	0 (0;0)
	Mean±SD	1.25±1.1	2.2±1.6	0.35±0.5	0.7±0.9	0.65±1.0	0.1±0.2
Total (all joints)	Median (25 p;75 p)	16.5 (15;20.5)	16(14;18)	3 (2;4.25)	6 (4;8.25)	5 (3;8)	0 (0;0)
	Mean±SD	18±6.1	16.8±5.3	3.9±3.5	6.6±3.4	5.5±3.2	0.2±0.5
RA patients							
Total PIP score	Median (25 p;75 p)	3 (2;7)	4 (3;6)	3 (1;5)	2 (0;4)	2 (1;4)	1 (0;3)
	Mean±SD	4.6±2.4	4.6±2.4	3.8±4.2	2.2±1.9	5.5±3.2	2.2±2.8
Total MCP score	Median (25 p;75 p)	12(8;13)	11(9;13)	5 (3;7)	4 (3;5)	3 (2;6)	2 (0;4)
	Mean±SD	11.0±3.1	11.2±3.4	6±5.1	4.8±5.1	4.9±4.8	3.8±6.15
Total Wrist Score	Median (25 p;75 p)	2 (1;4)	2 (1;4)	2 (1;3)	1 (0;3)	2 (1;4)	1 (0;2)
	Mean±SD	2.3±1.6	2.6±1.6	1.9±1.6	1.7±1.6	2.0±1.5	1.4±1.7
Total (all joints)	Median (25 p;75 p)	18(14;21)	19(15;21)	10(7;12)	7 (5;11)	7 (5;13)	5 (5;8)
	Mean±SD	17.8±5.6	18.4±5.2	11.8±7.8	8.7±5.9	9.5±7.3	7.6±8.4

Values are presented as median (25th percentile; 75th percentile) and mean (95% SD).

HUS, standard human ultrasonography; MCP, metacarpophalangeal; p, percentile; PIP, proximal interphalangeal (including 1st interphalangeal joint); RA, rheumatoid arthritis; RUS, robotic ultrasonography.

In healthy controls, RUS round 1 showed GS median sum score (range 0–66) was 16.5 (15; 20.5) and for CD 6 (4; 8.25), while for RUS round 2, GS median sum score was 16 (14; 18) and for CD 5 (3; 8) (table 2). The corresponding values for HUS were GS: 3 (2; 4.25) and CD: 0 (0;0) (table 2).

### Intrarobot agreement

The intrarobot agreement for patients with RA, based on GS and CD sum scores (range 0–66), showed a PEA of 10.3% for GS and 13.8% for CD. The PCA values were 37.9% for GS and 27.6% for CD, while ICC values were 0.65 for GS and 0.86 for CD in RA patients (table 3). For all joint pairs, the mean wKappa was 0.36 (SD=0.24) for GS and 0.35 (SD=0.24) for CD.

**Table 3:** Intrarobot agreement for RUS (RUS1 vs RUS2) for scores at joint level, joint region level and patient level for RA patients

Agreement RUS1 versus RUS2															
Baseline		Right hand				Left hand				Both hands					
		US GS		US CD		US GS		US DP		US GS			US CD		
		PEA	PCA	PEA	PCA	PEA	PCA	PEA	PCA	PEA	PCA	ICC	PEA	PCA	ICC
MCP	**1**	63.0	92.6	66.7	92.6	42.9	78.6	53.6	92.9	24.1	69.0		48.3	75.9	
	**2**	75.9	93.1	69.0	96.6	82.8	93.1	65.5	93.1	62.1	89.7		51.7	89.7	
	**3**	75.9	89.7	72.4	93.1	75.9	100	78.6	92.9	55.2	86.2		55.2	82.8	
	**4**	84.0	92.0	52.0	92.0	71.4	85.7	50.0	82.1	55.2	86.2		34.5	72.4	
	**5**	64.0	92.0	44.0	88.0	65.4	92.3	80.8	92.3	37.9	79.3		44.8	79.3	
	**Sum 1–5**	31.0	72.4	34.5	69.0	20.7	65.5	0.0	62.1	10.3	51.7	0.67	13.8	37.9	0.87
PIP	**1**	50.0	89.3	57.1	96.4	32.1	85.7	46.4	92.9	24.1	69.0		37.9	89.7	
	**2**	82.8	89.7	79.3	93.1	74.1	96.3	77.8	96.3	62.1	89.7		65.5	82.8	
	**3**	62.1	93.1	72.4	96.6	60.7	96.4	85.7	100	41.4	86.2		62.1	93.1	
	**4**	57.1	96.4	85.7	96.4	72.4	100	89.7	96.6	58.6	89.7		86.2	93.1	
	**5**	38.5	100	88.5	96.2	58.3	95.8	79.2	95.8	31.0	89.7		69.0	93.1	
	**Sum 1–5**	17.2	37.9	34.5	69.0	20.7	51.7	34.5	75.9	6.9	27.6	0.28	24.1	58.6	0.45
Wrist		58.6	93.1	34.5	69.0	50.0	85.7	46.4	92.9	34.5	82.8	0.58	44.8	86.2	0.81
Total		24.1	41.4	65.5	100	20.7	48.3	6.9	41.4	10.3	37.9	0.65	13.8	27.6	0.86

PEA expresses the percentage of the patients receiving the same score by RUS and PCA is the percentage of the patients where the scores differ no more than 1.0 between the two RUS scans. Intrarobot agreement on sum scores was assessed using single measure intraclass correlation coefficients (ICCs). An ICC ≥0.50 was considered moderate, an ICC ≥0.75 was considered good and an ICC ≥0.90 was considered excellent. Scoring ranges: joint level (0–3), joint region level (0–33), patient level (0.66).

CD, colour Doppler; GS, Greyscale; MCP, metacarpophalangeal; PCA, percentage of close agreement; PEA, percentage of exact agreement; PIP, proximal interphalangeal (including 1st interphalangeal joint); RUS, robotic ultrasonography; total, total sum joint score for both hands; US, ultrasound.

For healthy controls the intrarobot agreement based on GS and CD sum scores (range 0–66) was PEA of 5% for GS and 15% for CD. PCA values were 15% for GS and 40% for CD, while ICC values were 0.59 for GS and 0.64 for CD (table 4). The mean wKappa across all joint pairs was 0.41 (SD=0.21) for GS and 0.31 (SD=0.33) for CD.

**Table 4:** Intrarobot agreement for RUS (RUS1 vs RUS2) for scores at joint level, joint region level and patient level for healthy controls

Agreement RUS1 versus RUS2															
Baseline		Right hand				Left hand				Both hands					
		US GS		US CD		US GS		US DP		US GS			US CD		
		PEA	PCA	PEA	PCA	PEA	PCA	PEA	PCA	PEA	PCA	ICC	PEA	PCA	ICC
MCP	**1**	60.0	85.0	65.0	90.0	57.9	84.2	68.4	89.5	30.0	65.0		45.0	80.0	
	**2**	85.0	95.0	70.0	100.0	100	100.0	75.0	95.0	85.0	95.0		70.0	90.0	
	**3**	90.0	100	75.0	90.0	75.0	95.0	65.0	85.0	65.0	95.0		70.0	75.0	
	**4**	73.7	79.0	57.9	100.0	80.0	85.0	45.0	80.0	55.0	65.0		45.0	75.0	
	**5**	61.1	94.4	50.0	94.4	83.0	88.0	61.1	88.9	77.8	70.0		33.3	94.4	
	Sum 1–5	15.0	70.0	25.0	75.0	20.0	45.0	20.0	65.0	5.0	40.0	0.38	25.0	70.0	0.50
PIP	1	63.1	94.7	68.4	94.7	61.1	94.4	64.7	100.0	47.4	73.7		66.7	88.9	
	2	75.0	90.0	100.0	100.0	65.0	85.0	85.0	100.0	55.0	75.0		85.0	100.0	
	3	55.0	90.0	95.0	95.0	80.0	90.0	95.0	100.0	50.0	75.9		95.0	95.0	
	4	68.4	100	89.5	100.0	70.0	95.0	100.0	100.0	50.0	95.0		85.0	100.0	
	5	52.6	94.7	89.5	94.7	45.0	95.0	80.0	100.0	31.0	89.7		75.0	90.0	
	Sum 1–5	30.0	60.0	50.0	90.0	10.0	55.0	45.0	90.0	10.0	45.0	0.66	40.0	85.0	0.74
Wrist		40.0	75.0	70.0	90.0	70.0	95.0	70.0	95.0	40.0	70.0	0.41	50.0	85.0	0.48
Total		5.0	20.0	5.0	55.0	0	20.0	20.0	50.0	5.0	15.0	0.59	15.0	40.0	0.64

PEA expresses the percentage of the patients receiving the same score by RUS and PCA is the percentage of the patients where the scores differ no more than 1.0 between the two RUS scans. Intrarobot agreement on sum scores was assessed using single measure intraclass correlation coefficients (ICCs). An ICC ≥0.50 was considered moderate, an ICC ≥0.75 was considered good and an ICC ≥0.90 was considered excellent. Scoring ranges: joint level (0–3), joint region level (0–33), patient level (0.66).

CD, colour Doppler; GS, greyscale; MCP, metacarpophalangeal ; PCA, percentage of close agreement; PEA, percentage of exact agreement; PIP, proximal interphalangeal (including 1st interphalangeal joint); RUS, robotic ultrasonography; total, total sum joint score for both hands; US, ultrasound.

At the joint-level intra-robot agreement for GS (range 0–3) showed different PEA values according to joint type ranging between 32.1% and 82.8%, with a mean of 63.5%. The highest PEA values were observed in MCP2–4 and PIP2–4 joints (57.1%–84%), while the lowest agreement was found in the IP joint (32.1%–50%). CD agreement was relatively consistent across most joints, except for PIP4, which showed the highest PEA at 86.2%, while MCP4 (35.4%) and IP joints (37.9%) demonstrated the lowest values. PCA values for both GS and CD were similar across all joint levels (table 3). The agreement was slightly lower in healthy controls with a mean of PEA for GS 53.3% and CD at 61.5% and similar for PCA (table 4).

### Human-robot agreement

The overall human-robot agreement when based in the GS sum score (0–66) in patients with RA had PEA of 0%, PCA of 9.7% and an ICC of 0.59 for RUS round 1 versus HUS, while PEA 3.4%, PCA 6.9% and ICC 0.54 for RUS round 2 versus HUS. The corresponding CD values for RUS round 1 and RUS round 2 versus HUS were PEA 12.9% and 10.3%, PCA 32.3% and 27.9% and ICC 0.64 and 0.75, respectively, please see table 5. Comparable values were observed for healthy controls (table 6).

**Table 5:** Human-robot agreement between RUS (RUS1 and RUS2) and standard US (HUS) for baseline scores at joint level, joint region level and patient level for RA patients.

Baseline		Right hand				Left hand				Both hands					
		US GS		US CD		US GS		US CD		US GS			US CD		
		PEA	PCA	PEA	PCA	PEA	PCA	PEA	PCA	PEA	PCA	ICC	PEA	PCA	ICC
Agreement with HUS for RUS 1															
MCP	1	42.9	82.1	60.7	92.9	37.9	79.3	65.5	96.6	27.6	51.7		41.4	82.8	
	2	44.8	89.7	55.2	89.7	44.8	96.6	72.4	86.2	20.7	65.5		48.3	72.4	
	3	34.5	96.6	62.1	86.2	48.3	100.0	72.4	93.1	37.9	72.4		44.8	75.9	
	4	16.7	91.7	50.0	91.7	35.7	96.4	64.3	85.7	24.1	48.3		34.5	65.5	
	5	40.0	84.0	52.0	88.0	33.3	92.6	74.1	92.6	31.0	75.9		48.3	75.9	
	Sum 1–5	6.9	13.8	17.2	37.9	13.8	31.0	27.6	51.7	0.0	3.4	0.61	3.4	31.0	0.70
PIP	1	48.3	79.3	65.5	93.1	34.5	89.7	44.8	72.4	24.1	65.5		41.4	58.6	
	2	58.6	75.9	72.4	89.7	51.7	86.2	72.4	96.6	41.4	75.9		58.6	79.3	
	3	72.4	89.7	69.0	86.2	48.3	86.2	79.3	93.1	44.8	75.9		62.1	79.3	
	4	42.9	96.4	89.3	92.9	62.1	96.6	82.8	96.6	48.3	86.2		75.9	93.1	
	5	59.3	96.3	81.5	96.3	42.3	96.2	76.9	96.2	37.9	79.3		69.0	96.6	
	Sum 1–5	13.8	48.3	41.4	62.1	20.7	65.5	27.6	65.5	10.3	31.0	0.29	13.8	37.9	0.36
Wrist		37.9	89.7	65.5	86.2	32.1	96.4	64.3	96.4	13.8	58.6	0.49	41.4	79.3	0.71
Total		3.4	13.8	17.2	44.8	6.9	10.3	10.3	44.8	0.0	6.9	0.61	13.8	34.5	0.66
Agreement with HUS for RUS 2															
MCP	1	39.3	85.7	67.9	85.7	46.4	85.7	53.6	89.3	24.1	69.0		51.7	72.4	
	2	51.7	93.1	62.1	82.8	77.8	88.9	62.1	89.7	31.0	65.5		51.7	75.9	
	3	24.1	93.1	69.0	93.1	53.6	89.3	71.4	96.4	17.2	75.9		58.6	75.9	
	4	15.4	84.6	51.9	88.9	58.6	93.1	65.5	79.3	20.7	34.5		44.8	69.0	
	5	42.3	84.6	66.7	88.9	30.8	88.5	89.3	96.4	34.5	69.0		62.1	86.2	
Sum	1–5	0.0	20.7	24.1	55.2	3.4	31.0	20.7	62.1	0.0	6.9	0.49	13.8	27.6	0.78
PIP	1	21.4	85.7	60.7	89.3	46.4	85.7	42.9	75.0	27.6	69.0		20.7	72.4	
	2	69.0	89.7	82.8	89.7	77.8	88.9	77.8	92.6	55.2	75.9		75.9	93.1	
	3	75.9	96.6	62.1	89.7	53.6	89.3	78.6	92.9	44.8	89.7		65.5	89.7	
	4	48.3	93.1	82.1	89.3	58.6	93.1	86.2	93.1	41.4	86.2		75.9	86.2	
	5	60.7	96.4	85.2	92.6	30.8	88.5	69.2	92.3	27.6	79.3		65.5	86.2	
	Sum 1–5	20.7	41.4	31.0	65.5	20.7	55.2	24.1	62.1	10.3	31.0	0.53 (0.10;0.60	6.9	51.7	0.42
Wrist		37.9	79.3	65.5	93.1	44.8	89.7	51.7	96.6	31.0	55.2	0.63	44.8	82.8	0.85
Total	All	3.4	17.1	10.9	28.2	3.4	17.2	6.5	32.6	3.4	6.9	0.53	10.3	27.6	0.75

PEA expresses the percentage of the patients receiving the same score by RUS (RUS 1 and RUS 2) and HUS and PCA is the percentage of the patients where the scores differ no more than 1.0 between the RUS (RUS 1 and RUS 2) and HUS. Human-robot agreement on sum scores was assessed using single measure intraclass correlation coefficients (ICCs). An ICC ≥0.50 was considered moderate, an ICC ≥0.75 was considered good and an ICC ≥0.90 was considered excellent. Scoring ranges: joint level (0–3), joint region (0–33), patient level (0.66).

CD, colour Doppler; GS, greyscale; HUS, human ultrasonography; MCP, metacarpophalangeal; PCA, percentage of close agreement; PEA, percentage of exact agreement; PIP, proximal interphalangeal (including 1st interphalangeal joint); RA, rheumatoid arthritis; RUS, robotic ultrasonography; total, total sum joint score for both hands; US, ultrasound.

**Table 6:** Human-robot agreement between RUS (RUS1 respectively RUS2) and standard US (HUS) for baseline scores at joint level, joint region level and patient level for healthy controls.

Baseline		Right hand				Left hand				Both hands					
		US GS		US CD		US GS		US CD		US GS			US CD		
		PEA	PCA	PEA	PCA	PEA	PCA	PEA	PCA	PEA	PCA	ICC	PEA	PCA	ICC
Agreement with HUS for RUS 1															
MCP	1	15.0	50.0	75.0	95.0	35.0	75.0	85.0	95.0	10.0	30.0		65.0	85.0	
	2	40.0	85.0	80.0	95.0	25.0	100.0	85.0	90.0	15.0	45.0		70.0	90.0	
	3	35.0	95.0	90.0	90.0	35.0	85.0	70.0	90.0	20.0	55.0		65.0	80.0	
	4	5.3	78.9	47.4	89.5	45.0	85.0	55.0	90.0	5.0	35.0		35.0	65.0	
	5	33.3	94.4	77.8	100.0	27.8	88.9	88.9	94.4	22.2	44.4		66.7	94.4	
	Sum 1–5	5.0	10.0	30.0	60.0	5.0	20.0	20.0	50.0	0.0	10.0	0.05	10.0	25.0	0.06
PIP	1	63.2	89.5	63.2	89.5	55.0	95.0	65.0	95.0	42.1	68.4		36.8	84.2	
	2	70.0	90.0	95.0	100.0	65.0	90.0	100.0	100.0	60.0	70.0		95.0	100.0	
	3	60.0	85.0	100.0	100.0	80.0	95.0	95.0	100.0	60.0	70.0		95.0	100.0	
	4	78.9	100.0	94.7	100.0	70.0	95.0	100.0	100.0	50.0	95.0		95.0	100.0	
	5	57.9	89.5	84.2	94.7	35.0	85.0	70.0	95.0	15.0	60.0		70.0	85.0	
	Sum 1–5	10.0	40.0	50.0	80.0	20.0	45.0	50.0	85.0	5.0	15.0	0.30	25.0	70.0	0.07
Wrist		35.0	85.0	70.0	85.0	35.0	95.0	75.0	100.0	35.0	70.0	0.13	60.0	80.0	0.06
Total		0.0	5.0	21.1	21.1	0.0	5.0	10.0	25.0	0.0	0.0	0.30	10.0	15.0	0.06
Agreement with HUS for RUS 2															
MCP	1	25.0	75.0	90.0	95.0	36.8	89.5	61.1	88.9	15.0	40.0		65.0	90.0	
	2	40.0	90.0	85.0	100.0	25.0	100.0	85.0	100.0	10.0	50.0		65.0	95.0	
	3	35.0	95.0	80.0	95.0	35.0	90.0	90.0	100.0	15.0	60.0		60.0	80.0	
	4	20.0	90.0	45.0	75.0	40.0	100.0	100.0	100.0	20.0	40.0		25.0	45.0	
	5	26.3	89.5	63.2	94.7	31.6	84.2	80.0	95.0	11.1	44.4		33.3	83.3	
Sum	1–5	5.0	5.0	30.0	55.0	5.0	15.0	15.0	25.0	5.0	5.0	0.40	5.0	15.0	0.06
PIP	1	65.0	95.0	65.0	95.0	50.0	88.9	73.7	94.7	55.0	70.0		50.0	80.0	
	2	95.0	100.0	95.0	100.0	70.0	95.0	75.0	100.0	70.0	90.0		80.0	100.0	
	3	80.0	95.0	95.0	95.0	90.0	95.0	65.0	85.0	75.0	95.0		90.0	95.0	
	4	90.0	100.0	90.0	100.0	90.0	90.0	40.0	75.0	80.0	90.0		90.0	100.0	
	5	55.0	95.0	90.0	100.0	40.0	95.0	63.2	84.2	25.0	65.0		75.0	95.0	
	Sum 1–5	35.0	65.0	60.0	75.0	10.0	55.0	50.0	75.0	5.0	15.0	0.37 (0.10;0.60	40.0	50.0	0.12
Wrist		20.0	70.0	65.0	90.0	25.0	85.0	80.0	90.0	20.0	40.0	0.13	60.0	80.0	0.03
Total	All	0.0	0.0	15.0	30.0	0.0	5.0	5.0	20.0	0.0	0.0	0.57	5.0	5.0	0.14

PEA expresses the percentage of the patients receiving the same score by RUS (RUS 1 and RUS 2) and HUS and PCA is the percentage of the patients where the scores differ no more than 1.0 between the RUS (RUS 1 and RUS 2) and HUS. Human-robot agreement on sum scores was assessed using single measure intraclass correlation coefficients (ICCs). An ICC ≥0.50 was considered moderate, an ICC ≥0.75 was considered good and an ICC ≥0.90 was considered excellent. Scoring ranges: joint level (0–3), joint region (0–33), patient level (0.66).

CD, colour Doppler; GS, greyscale; HUS, human ultrasonography; MCP, metacarpophalangeal; PCA, percentage of close agreement; PEA, percentage of exact agreement; PIP, proximal interphalangeal (including 1st interphalangeal joint); RUS, robotic ultrasonography; total, total sum joint score for both hands; US, Ultrasound.

In terms of human-robot agreement at the joint level, the PEA for GS ranged from 15.4% to 77.8% (mean: 45.6%), while CD ranged from 42.9% to 90.0% (mean: 66.9%). The lowest GS agreement was observed in the MCP and wrist joints (range: 12.9%–51.7%). CD consistently showed higher agreement across all joint regions than GS (table 5). Similarly, this was seen among healthy controls. There, a low GS agreement was seen at the MCP joint level, with mean PEA values of 14.4% for RUS1 and 14.2% for RUS2. Wrist agreement was slightly higher, at 27.5% (table 6).

### Diagnostic accuracy, sensitivity and specificity

The diagnostic sensitivity, specificity and accuracy for HUS for identifying patients with clinically detected arthritis (reference standard) was 100%, 95% and 98%, respectively. For RUS round 1, a sensitivity of 60%, specificity of 5% and an accuracy of 59% were demonstrated. The PEA between RUS round 1 and HUS was 59%, with a Cohen’s Kappa of 0.02. Similarly, RUS round 2 showed a sensitivity of 58%, specificity of 0% and an accuracy of 59%. The PEA with HUS was 57%, with a Kappa value of −0.04.

### Examination time

The median scanning time for a single RUS examination (including synovitis scoring) was 18 min and 50 s (range: 16:48–20:38), while the median scan time for HUS was 12 min and 8 s (range: 10:49–13:49). Additionally, approximately 2–3 min should be added for entering the HUS synovitis scores.

### Failed joint assessments

In healthy controls, RUS round 1 failed to assess 1.6% (7/440) of joints and 1.1% (5/440) in RUS round 2. Among RA patients, 2.8% (18/638) and 2.5% (16/638) of joints were not assessable in rounds 1 and 2, respectively. The corresponding median HUS score for these joints was 0 (range 0–2). Notably, 98% and 97% of all failed assessments in rounds 1 and 2, respectively, were localised to MCP4, MCP5 and PIP5. There were no adverse events observed during the study.

## Discussion

This study is the first to present a validation of a US-based robotic system with an AI-driven solution for synovitis assessment in RA patients and healthy controls. We demonstrated a moderate to good agreement between the RUS scans in both RA patients and healthy controls, as measured by ICC, and slightly lower agreement when evaluated using wKappa. Though this level of agreement is not impressive, we consider it acceptable, as it is only marginally lower than what has been observed between expert assessors^10^ suggesting the performance of the robot is at the level of another HUS but not better. Our findings highlight a significant potential for RUS, particularly in regions with limited rheumatologists’ availability, as RUS offers clear advantages for arthritis assessment over other low-cost imaging modalities such as fluorescence optical imaging and optical spectral transmission. Unlike these techniques, which primarily reflect changes in blood flow, RUS provides detailed anatomical information about the joints and location of the inflammation and may run with limited assistance from healthcare professionals.^11 17^

Nevertheless, this discrepancy highlights an important focus area for future development, especially considering that robotic and AI-based technologies have the potential to outperform human assessments.^18 19^ For synovitis scores, RUS yielded higher sum GS MCP scores (mean=11.2) compared with HUS (mean=6.0), while scores for PIP and wrist joints were only slightly higher in RUS. The total CD mean score for RUS was also higher than HUS, with a mean difference of 1.5, driven across all joint regions. A similar trend was observed in healthy controls. These findings were unexpected, particularly the high MCP scores observed in healthy controls, given that we had previously demonstrated a moderate to good total correlation between RUS and HUS in RA patients. Therefore, in collaboration with the company behind the ARTHUR system, we reviewed all settings and images and identified that the overall mean gain was lower than previously tested by the company. However, this discrepancy was not visually apparent during manual image review in most cases. The lower gain may very well have contributed to a higher DIANA score, as the synovium becomes more difficult to delineate for the system. Despite a thorough investigation of all settings, it was not possible to determine the cause of the lower gain when the images were read by DIANA. It is well-known that ultrasound beam reflection varies depending on human tissue, which can cause slight differences in gain despite identical US machine settings.^20^ Unfortunately, the current version of DIANA cannot adjust for gain differences.

We also observed that the CD scores were higher for RUS than for HUS. This was unexpected, as the ARTHUR system applies slightly more consistent pressure throughout the examination than a human assessment. The higher scores could potentially be explained by Doppler reverberation and motion artefacts,^21^ which may have been mistakenly interpreted as true signals by DIANA. Reverberation and motion artefacts were observed in a minority of images on inspection. While such artefacts can easily be identified when the ultrasound examination is performed by a rheumatologist, this may be more challenging for DIANA. Improved artefact recognition should ideally be incorporated into future versions of the system.

The agreement between RUS and HUS in detecting synovitis at patient level demonstrated a moderate to good overall agreement, measured by ICC in patients with RA with the best reliability seen for CD, indicating that Doppler US performs better in an AI-based solution than GS US, as identifying colour is easier than identifying different shades of grey and thus more straightforward to implement in AI-based solutions. However, at joint level, low PEA was observed, particularly for GS scoring of MCP joints. This was expected, due to the observed higher MCP joint score for RUS. This low agreement is important to pay attention to, as it is well established that AI algorithms tend to pick up and repeat hidden biases in the data they are trained on.^22^ For example, a slightly reduced gain setting during image acquisition can significantly alter the model’s interpretation, leading to a disproportionately large impact on the outcome, as most likely seen in this study.

Finally, we evaluated the diagnostic performance of RUS using the clinically detected arthritis as references, applying a moderate cut-off for US-detected arthritis,^9^ though alternative thresholds may be applied. Unfortunately, RUS demonstrated very low specificity and only moderate accuracy for clinically detected arthritis, which mainly is explained by the high MCP joint GS scores in healthy controls.

In this study, we evaluated the outcomes of combining robotic ultrasound systems for image acquisition with AI-based software for automated scoring. However, an alternative approach that remains unexplored is whether manual interpretation of robot-acquired images at the current ARTHUR version could yield better outcomes. We believe that manual reading of these images holds promise for clinical application. Further studies are needed to investigate this possibility.

Furthermore, we did not do joint-level setting adjustments during HUS. This may have affected its diagnostic performance. One of the strengths in this study is the inclusion of healthy controls and RA patients with active synovitis in the hands, which provides a perfect mimic of the clinical setting where RUS will be relevant. On the other hand, it is important to note that patients with severe hand deformities were excluded from the study, and approximately 2% of the examined joints could not be assessed by the ARTHUR system, mainly located at the joint level of MCP4, MCP5 and PIP5. It might be due to the gel’s tendency to slide off the skin. This may have positively influenced the overall performance.

Overall, automated synovitis assessment is, with the current ARTHUR/Diana version (1.5.1/2.0.1), not ready to be implemented in daily practice but has the potential to change how we manage rheumatology patients in the future. As the Robot and AI technology develop is it likely that automated ultrasound systems can provide consistent results by eliminating human variability in acquisition and interpretation. Furthermore, automated systems will potentially make ultrasound examinations more accessible, especially in areas with a shortage of rheumatologists, and potentially allowing a more sensitive diagnosis and potential monitoring of patients with RA.^6 23 24^

Continuous improvement in robots and software occurs, and the performance of future updated versions of ARTHUR and DIANA should be similarly explored with respect to optimised performance.

In conclusion, we have demonstrated an overall moderate to good agreement between RUS and HUS in assessing synovitis in RA hands when using ARTHUR, suggesting a potential clinical role. However, low agreement was seen at the joint level, particularly for MCP joints, both for the intrarobot agreement and human-robot agreement. Further development and software adjustments are needed.

## Data availability statement

Data are available upon reasonable request.

## References

[1] Finckh A, Gilbert B, Hodkinson B (2022). Global epidemiology of rheumatoid arthritis. Nat Rev Rheumatol.

[2] Safiri S, Kolahi AA, Hoy D (2019). Global, regional and national burden of rheumatoid arthritis 1990-2017: a systematic analysis of the Global Burden of Disease study 2017. Ann Rheum Dis.

[3] Aletaha D, Neogi T, Silman AJ (2010). 2010 Rheumatoid arthritis classification criteria: An American College of Rheumatology/European League Against Rheumatism collaborative initiative. Arthritis & Rheumatism.

[4] Hetland ML, Stengaard-Pedersen K, Junker P (2006). Combination treatment with methotrexate, cyclosporine, and intraarticular betamethasone compared with methotrexate and intraarticular betamethasone in early active rheumatoid arthritis: an investigator-initiated, multicenter, randomized, double-blind, parallel-group, placebo-controlled study. Arthritis Rheum.

[5] Colebatch AN, Edwards CJ, Østergaard M (2013). EULAR recommendations for the use of imaging of the joints in the clinical management of rheumatoid arthritis. Ann Rheum Dis.

[6] Ammitzbøll-Danielsen M, Fana V, Døhn UM (2022). Ultrasound assessment of hands and feet for synovitis at time of first clinical visit markedly reduces time to diagnosis in routine care. Rheumatology (Oxford).

[7] Stein M, Vaillancourt J, Rampakakis E (2020). Prospective observational study to evaluate the use of musculoskeletal ultrasonography in rheumatoid arthritis management: the ECHO study. Rheumatology (Oxford).

[8] Frederiksen BA, Schousboe M, Terslev L (2022). Ultrasound joint examination by an automated system versus by a rheumatologist: from a patient perspective. *Adv Rheumatol*.

[9] D’Agostino M-A, Terslev L, Aegerter P (2017). Scoring ultrasound synovitis in rheumatoid arthritis: a EULAR-OMERACT ultrasound taskforce**-**Part 1: definition and development of a standardised, consensus-based scoring system. RMD Open.

[10] Terslev L, Naredo E, Aegerter P (2017). Scoring ultrasound synovitis in rheumatoid arthritis: a EULAR-OMERACT ultrasound taskforce-Part 2: reliability and application to multiple joints of a standardised consensus-based scoring system. RMD Open.

[11] Ammitzbøll-Danielsen M, Glinatsi D, Terslev L (2022). A novel fluorescence optical imaging scoring system for hand synovitis in rheumatoid arthritis-validity and agreement with ultrasound. Rheumatology (Oxford).

[12] Ammitzbøll-Danielsen M, Østergaard M, Naredo E (2016). Validity and sensitivity to change of the semi-quantitative OMERACT ultrasound scoring system for tenosynovitis in patients with rheumatoid arthritis. Rheumatology (Oxford).

[13] Wells G, Becker J-C, Teng J (2009). Validation of the 28-joint Disease Activity Score (DAS28) and European League Against Rheumatism response criteria based on C-reactive protein against disease progression in patients with rheumatoid arthritis, and comparison with the DAS28 based on erythrocyte sedimentation rate. Ann Rheum Dis.

[14] Koo TK, Li MY (2016). A Guideline of Selecting and Reporting Intraclass Correlation Coefficients for Reliability Research. J Chiropr Med.

[15] Light RJ (1971). Measures of response agreement for qualitative data: Some generalizations and alternatives. Psychol Bull.

[16] Dettori JR, Norvell DC (2020). Kappa and Beyond: Is There Agreement?. Global Spine J.

[17] Krabbe S, Ammitzbøll-Danielsen M, Østergaard M (2016). Sensitivity and specificity of optical spectral transmission imaging in detecting joint inflammation in rheumatoid arthritis. Ann Rheum Dis.

[18] Jaruvongvanich V, Muangsomboon K, Teerasamit W (2024). Optimizing computed tomography image reconstruction for focal hepatic lesions: Deep learning image reconstruction vs iterative reconstruction. Heliyon.

[19] Paudyal R, Shah AD, Akin O (2023). Artificial Intelligence in CT and MR Imaging for Oncological Applications. Cancers (Basel).

[20] Quarato CMI, Lacedonia D, Salvemini M (2023). A Review on Biological Effects of Ultrasounds: Key Messages for Clinicians. Diagnostics (Basel).

[21] Terslev L, Diamantopoulos AP, Døhn UM (2017). Settings and artefacts relevant for Doppler ultrasound in large vessel vasculitis. *Arthritis Res Ther*.

[22] Koçak B, Ponsiglione A, Stanzione A (2025). Bias in artificial intelligence for medical imaging: fundamentals, detection, avoidance, mitigation, challenges, ethics, and prospects. Diagn Interv Radiol.

[23] D’Agostino MA, Terslev L, Wakefield R (2016). Novel algorithms for the pragmatic use of ultrasound in the management of patients with rheumatoid arthritis: from diagnosis to remission. Ann Rheum Dis.

[24] Naredo E, Valor L, De la Torre I (2015). Predictive value of Doppler ultrasound-detected synovitis in relation to failed tapering of biologic therapy in patients with rheumatoid arthritis. Rheumatology (Oxford).

